# Drop-out from the tuberculosis contact investigation cascade in a routine public health setting in urban Uganda: A prospective, multi-center study

**DOI:** 10.1371/journal.pone.0187145

**Published:** 2017-11-06

**Authors:** Mari Armstrong-Hough, Patricia Turimumahoro, Amanda J. Meyer, Emmanuel Ochom, Diana Babirye, Irene Ayakaka, David Mark, Joseph Ggita, Adithya Cattamanchi, David Dowdy, Frank Mugabe, Elizabeth Fair, Jessica E. Haberer, Achilles Katamba, J. Lucian Davis

**Affiliations:** 1 Uganda Tuberculosis Implementation Research Consortium, Makerere University, Kampala, Uganda; 2 Department of Epidemiology of Microbial Diseases, Yale School of Public Health, New Haven, Connecticut, United States of America; 3 Division of Pulmonary and Critical Care Medicine, University of California San Francisco, San Francisco, California, United States of America; 4 Bloomberg School of Public Health, Johns Hopkins University, Baltimore, Maryland, United States of America; 5 National Tuberculosis & Leprosy Programme, Uganda Ministry of Health, Kampala, Uganda; 6 Massachusetts General Hospital, Boston, Massachusetts, United States of America; 7 Clinical Epidemiology Unit, Makerere University, Kampala, Uganda; 8 Pulmonary, Critical Care, and Sleep Medicine Section, Yale School of Medicine, New Haven, Connecticut, United States of America; University of New South Wales, AUSTRALIA

## Abstract

**Setting:**

Seven public tuberculosis (TB) units in Kampala, Uganda, where Uganda’s national TB program recently introduced household contact investigation, as recommended by 2012 guidelines from WHO.

**Objective:**

To apply a cascade analysis to implementation of household contact investigation in a programmatic setting.

**Design:**

Prospective, multi-center observational study.

**Methods:**

We constructed a cascade for household contact investigation to describe the proportions of: 1) index patient households recruited; 2) index patient households visited; 3) contacts screened for TB; and 4) contacts completing evaluation for, and diagnosed with, active TB.

**Results:**

338 (33%) of 1022 consecutive index TB patients were eligible for contact investigation. Lay health workers scheduled home visits for 207 (61%) index patients and completed 104 (50%). Among 287 eligible contacts, they screened 256 (89%) for symptoms or risk factors for TB. 131 (51%) had an indication for further TB evaluation. These included 59 (45%) with symptoms alone, 58 (44%) children <5, and 14 (11%) with HIV. Among 131 contacts found to be symptomatic or at risk, 26 (20%) contacts completed evaluation, including five (19%) diagnosed with and treated for active TB, for an overall yield of 1.7%. The cumulative conditional probability of completing the entire cascade was 5%.

**Conclusion:**

Major opportunities exist for improving the effectiveness and yield of TB contact investigation by increasing the proportion of index households completing screening visits by lay health workers and the proportion of at-risk contacts completing TB evaluation.

## Introduction

Household contact investigation, a systematic approach to identifying undiagnosed TB patients among close contacts of confirmed TB patients[1], was first endorsed by WHO for routine implementation in low- and middle-income countries in 2012.[2] However, information about the performance of contact investigation for identifying new patients with active TB and initiating them on treatment is limited. Specifically, there are few details about when and where participants are lost-to-follow-up, essential information for designing interventions to improve delivery of contact investigation.[3–5] Cascade analysis, frequently applied to understand cumulative losses along the HIV care continuum[6,7], can clarify the most important gaps in delivery of TB evaluation and care, and in so doing inform the design and targeting of interventions.[8]

Identifying TB patients via household contact investigation depends on effectively completing four key steps in the delivery cascade: 1) scheduling visits to homes of index TB patients; 2) initiating these visits by physically reaching the homes of index patients; 3) screening all household contacts; and 4) ensuring that all symptomatic and high-risk contacts complete TB evaluation. Previously, we identified several barriers to participants completing these steps, including a lack of knowledge about TB and its potential consequences; HIV and TB stigma; mistrust of health workers; and insufficient time and resources.[9,10]

In 2013, the Uganda National TB and Leprosy Programme (NTLP) began implementing household TB contact investigation for all index TB patients in the country’s capital, Kampala. Given the above-noted evidence gaps, we launched a prospective, observational study to determine the effectiveness of each step of contact investigation and its overall yield using a cascade analysis.

## Study population and methods

### Setting and study design

The study took place at six public primary-health centers and one general hospital in Kampala, Uganda, from September 2015 to July 2016. All seven sites offer TB diagnostic and treatment services to residents of Greater Kampala in specialized TB units, and had implemented contact investigation as part of a new program led by the NTLP in partnership with a non-governmental organization. This study was designed to describe the implementation of contact investigation under programmatic conditions. The NTLP trained lay health workers to maintain the TB register and carry out household contact investigation; study staff trained lay health workers to document the contact investigation cascade using tablet computers during weekly supervisory visits to each site. Study staff also carried out audits of these data to ensure accuracy.

### Study population

The study population included consecutive index patients treated for drug-susceptible pulmonary TB (microbiological confirmation required for patients ≥5 years) and their household contacts. Index patients were included if they resided in Greater Kampala and reported having household contacts, and were excluded if they did not have access to a mobile phone, were unable to speak English or Luganda, or did not consent. Household contacts were included if they slept under the same roof as the index patient ≥5 nights within the previous three months and were not currently receiving TB treatment, and were excluded if they did not have access to a mobile phone, did not consent, or did not speak English or Luganda.

### Enrollment and baseline data collection

Lay health workers reviewed all index patients at treatment initiation, consenting and enrolling eligible index patients using tablet computers equipped with an open-source application for data capture (CommCare, Dimagi, Cambridge, MA, USA) and customized for fingerprint-recognition (Biometrac, Louisville, KY, USA). At enrollment, lay health workers collected demographic and clinical data from index patients and scheduled household visits. If a patient preferred to schedule the visit by phone, lay heath workers made at least three calls, and, if unsuccessful, attempted to arrange the household visit at the index patient’s two-week follow-up visit. Several months into enrollment, lay health workers began asking index patients why they declined consent and systematically documenting the circumstances surrounding unsuccessful home visits.

To identify contacts, lay health workers reviewed all individuals present at the household visit for eligibility. If one or more reported contacts were not available at the initial visit, lay health workers scheduled additional household visits in an effort to reach all possible contacts. After verifying eligibility and obtaining consent, lay health workers provided basic TB education and counseling; collected demographic information, fingerprints, and clinical data; and instructed contacts with TB symptoms or risk factors (*i*.*e*., HIV-seropositivity, age <5) to attend one of the seven TB units for further testing and evaluation. In accordance with local policy for household contact investigation, lay health workers gave at-risk contacts written referrals for clinical evaluation, allowing them to proceed directly to a TB unit for expedited evaluation. There, lay health workers confirmed each contact’s identity and laboratory results by name or fingerprint against electronic tablet rosters before proceeding with TB evaluation and treatment. Additional details appear in the S1 File supplement.

### Definitions

We defined four key steps of the contact investigation cascade: Step 1) Home visit scheduled—Proportion of eligible index patients for whom health workers confirmed a date and time for a home visit; Step 2) Home visit initiated–Proportion of scheduled home visits where ≥1 possible contact was encountered; Step 3) Contacts screened–Proportion of eligible contacts who completed assessment of TB symptoms and risk factors; Step 4) Contacts completed TB evaluation–Proportion of contacts with TB symptoms and/or risk factors found during TB screening (child <5 or HIV-seropositive) who complete follow-up medical evaluation. We defined completion of medical evaluation as either (1) one positive or negative Xpert result, (2) one positive or two negative sputum-smear results, or (3) documented clinical evaluation for TB (required for children <5 and those with HIV).

Because drop-out can occur at any point in the contact investigation cascade, we defined drop-out among eligible index patients or household contacts as failure to move from any defined step above to the subsequent step, when eligible. We defined effectiveness as the cumulative probability of a household contact with symptoms or risk factors completing all steps in the cascade, including follow-up evaluation for TB. Finally, we defined yield as standardly defined in the contact investigation literature[5,11]: the proportion of eligible contacts encountered during baseline contact investigation who were diagnosed with TB as a result of contact investigation.

### Analytic methods

We constructed a cascade tracing the flow of index patients and their household contacts through each step of contact investigation. We calculated descriptive statistics for participant characteristics and the proportion of participants completing each step of the contact investigation cascade, from the identification of eligible households through follow-up medical evaluation of household contacts found to be symptomatic or at risk for TB. We corrected for clustering of symptoms, risk factors, and follow-up behavior by household by calculating robust standard errors using generalized estimation equations (GEE) in SAS University Edition (SAS Institute, Cary, NC). We estimated the cumulative probability of a contact with TB symptoms or risk factors completing contact investigation by multiplying the proportions completing each of the four major steps of the cascade as conditional probabilities, assuming similar household characteristics among those retained and those lost. We estimated the yield of contact investigation as the proportion of encountered, eligible contacts diagnosed with TB with exact binomial 95% confidence intervals, and its reciprocal, the number-needed-to-screen.

### Protection of human subjects

Each participant and/or a parent or guardian provided written informed consent and/or assent. The School of Medicine Research and Ethics Committee at the Makerere College of Health Sciences, the Uganda National Council for Science and Technology, and the Human Investigation Committee at Yale University approved the study.

## Results

### Screening of index TB patients

**Fig 1:**
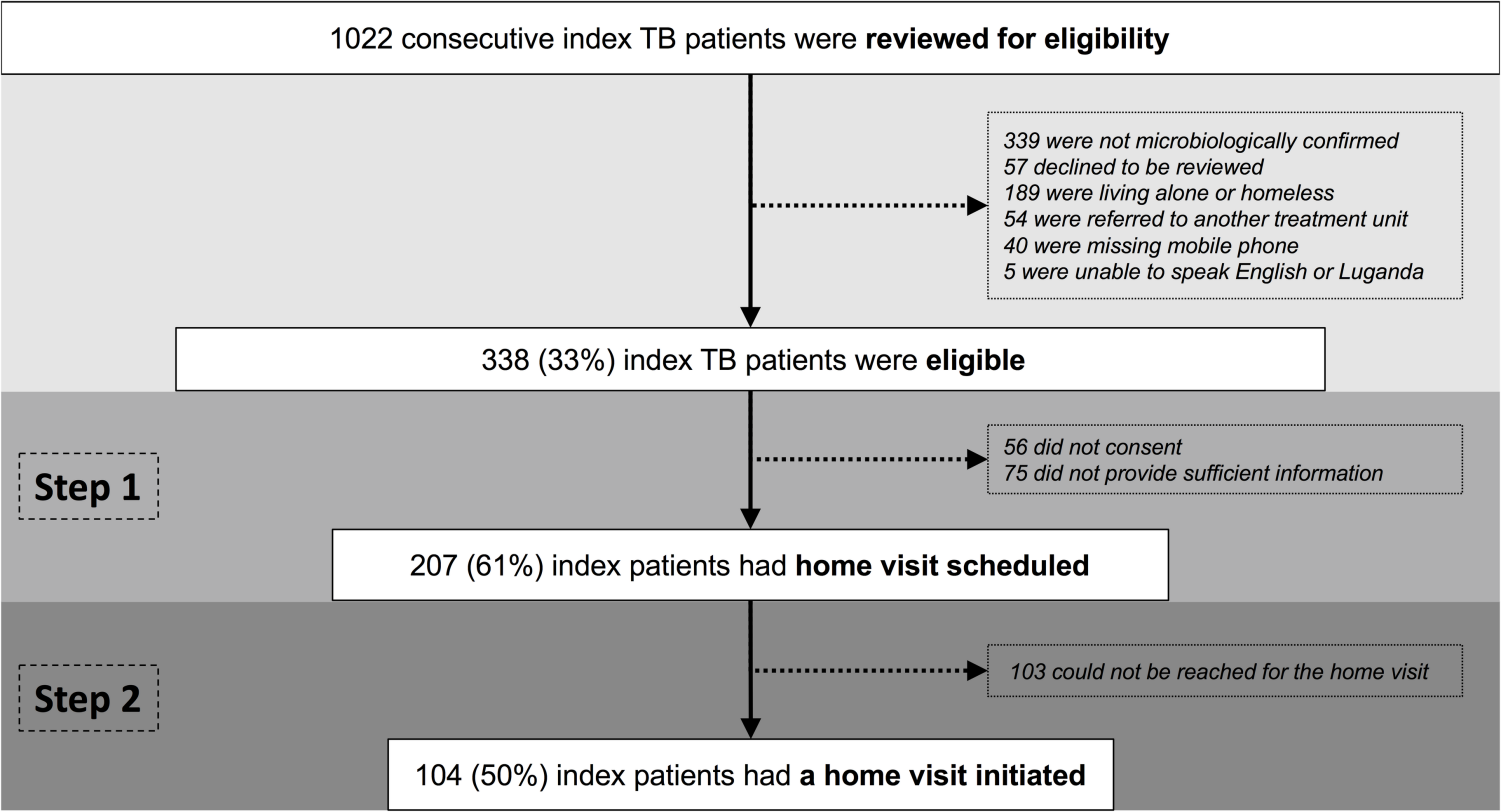
Flow diagram describing the evaluation cascade for index TB patients. The figure presents the steps of the evaluation cascade for index TB patients, with the target population identified in bold font. All percentages are calculated as a proportion of the number of participants entering the previous step of the cascade. Abbreviations: CI, contact investigation; MDR, multi-drug-resistant; TB, tuberculosis.

Among 1022 consecutive index TB patients, contact investigation was not indicated in 585 (57%), including 339 lacking microbiological confirmation; 57 declining to be reviewed for eligibility for contact investigation; and 189 living alone or homeless. Fifty-four (5%) coming from outside Kampala were referred to another treatment unit near their homes, and the remaining 45 were inaccessible to health workers, including 40 without access to a mobile phone, and five unable to speak English or Luganda (Fig 1). This left 338 (33%) eligible for household contact investigation.

### Step 1 and Step 2: Scheduling and initiating home visits

Lay health workers successfully scheduled home visits for 207 of 338 (61%) eligible index patients (range across health centers, 18–71%). Reasons for not scheduling home visits included lack of consent (n = 56) or insufficient contact information from the index patient (n = 75). The most common reason for not consenting was not having “enough time” to participate (12, 43%; Table C in S1 File). Lay health workers completed 104 (50%) of the 207 home visits scheduled (range across health centers, 11–100%). The most common reasons for failed home visits were index patients moving out of the home or contacts expressing unwillingness to participate in contact investigation to the index patient. Median time between diagnosis of the index patient and screening of household contacts for TB was nine days (interquartile range, 1–18 days).

### Characteristics of index patients and their households

Of 104 index patients whose households were visited, 53 were men (56%; demographic and clinical data missing for nine patients) and 29 (31%) were HIV-positive (Table 1). Index patients reported coughing for a median of four weeks (range 1–52 weeks) before receiving a TB diagnosis. Six (6.3%) reported having more than one household, giving an estimated total of 110 reported households. The number of contacts per household ranged from one to 12 (median 2). Demographic and clinical characteristics were similar for the 103 index patients whose households were not reached (Table A in S1 File).

**Table 1:** Characteristics of index TB patients and their households.

Characteristic ^a^	n (%)
*Visited index TB patients (n = 104*^b^*)*	
Men	53 (56%)
HIV-seropositive	29 (31%)
Age groups	
Adults (≥15 years)	92 (97%)
Older children (5–14 years)	2 (2%)
Younger children (0–4 years)	1 (1%)
>1 household	6 (6%)
Number of contacts (range)	
Reported	3 (1–25)
Screened for TB	2 (1–12)
Cough duration, weeks (25^th^-75^th^ %ile)	8 (4–12)
Microbiologically confirmed	94 (99%)
*Index TB patient households (n = 101*^b^*)*	
Weekly income in USD (IQR)^c^	$15.40 ($5.60 - $28.10)
Weekly expenditure in USD (IQR)^d^	$8.40 ($4.20 - $19.60)
Number of rooms (range)	2 (1–10)
Literacy, head of household^e^	62 (95%^b^)
Underwent >1 visit to household	13 (12%)
Indications for TB evaluation^f^	
≥1 contact with TB symptoms	55 (51%)
≥1 child contact under 5	43 (40%)
≥1 HIV-seropositive contact	7 (7%)

Legend: Percentages may not sum to 100% due to rounding.

^a^ Table values are median (range or interquartile range, as specified) for continuous variables and n (column %) for categorical variables.

^b^ Due to missing demographic data for nine index patients, Table 1 describes an analytic sample of 95 index patients contributing 101 households.

^c^ Missing n = 30

^d^ Missing n = 52

^e^ Missing n = 30

^f^ Among contacts. Abbreviations: IQR, interquartile range; TB, tuberculosis; USD, US dollars

Lay health workers identified heads of household in 65 (65%) households. Of these, 62 (95%) were able to read and write, and median weekly household income was USD $15.40 (25^th^-75^th^ percentile, $5.60 - $28.10) (Table 1). Fifty-five households (51%) included at least one contact reporting a TB symptom, 43 (40%) included at least one child under age five, and 7 (6.5%) included at least one known HIV-seropositive contact.

### Identification of household contacts

**Fig 2:**
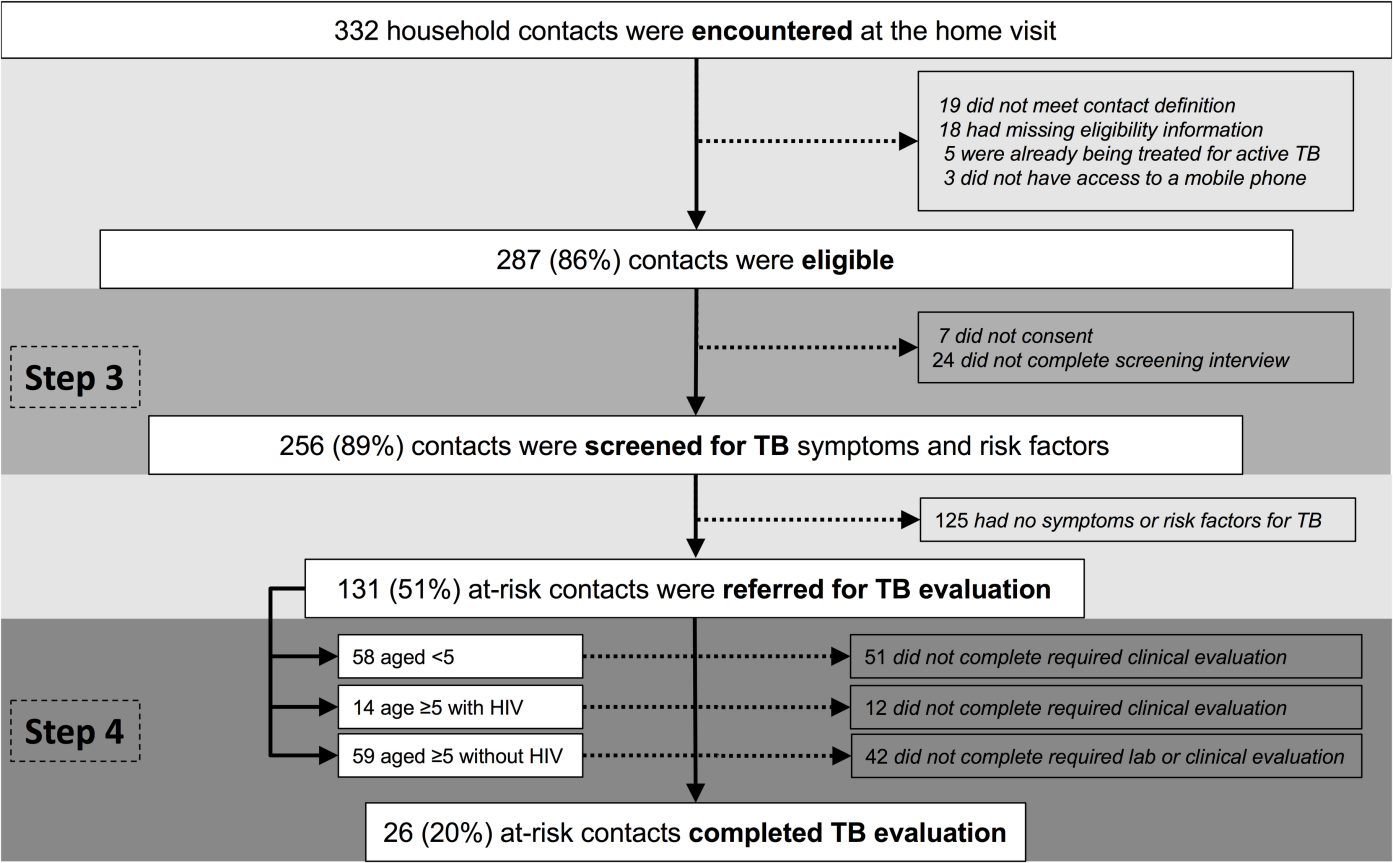
Flow diagram describing the evaluation cascade for household contacts. The figure presents the steps of the evaluation cascade for household contacts, with the target population identified in bold font below the pre-screening population. All percentages are calculated as a proportion of the number of participants entering the previous step of the cascade. Abbreviations: CI, contact investigation; TB, tuberculosis.

Index patients visited by lay health workers reported a total of 346 individuals living in their households, or 3.4 possible contacts per household. During visits, lay health workers actually encountered 332 possible household contacts, of whom 287 (86%) were eligible (Fig 2). Of these 287 eligible contacts, 24 (8%) did not complete the TB screening interview and seven (2%) did not consent, resulting in 256 (89%) household contacts who were screened for TB symptoms and risk factors.

### Characteristics of household contacts

Most (143/256, 56%) enrolled contacts were adults 15 years or older, but substantial proportions were older children aged 5–14 years (55, 22%) or younger children 0–4 years (58, 23%). Ninety-four contacts (37%) were male, and 14 (5.5%) reported being HIV-seropositive (Table 2).

**Table 2:** Characteristics of household contacts.

Characteristic^a^	Household Contacts
*n (%)*	*n = 256*
Male gender	94 (37%)
Age groups	
Adults (≥15 years)	143 (56%)
Older children (5–14 years)	55 (22%)
Younger children (0–4 years)	58 (23%)
Known HIV-seropositive	14 (6%)
Symptoms of TB	
Cough of any duration	88 (34%)
Cough ≥2 weeks	63 (25%)
Subjective fever	31 (12%)
Night sweats	29 (11%)
Weight loss	34 (13%)

Legend: Percentages may not sum to 100% due to rounding.

^a^ Table values are n (column %) for categorical variables.

Abbreviations: TB, tuberculosis.

### Step 3: Screening of household contacts for symptoms and risk factors of TB

Of 287 contacts found to be eligible, 256 (89%) were screened for symptoms and risk factors of TB (range across health centers, 55%-100%). About half of the 256 contacts screened for TB (131, 51%; robust 95% confidence limits, 44%-58%) had at least one indication for further TB evaluation, including 59 adults and older children who reported ≥1 TB symptom, all 58 younger children, and all 14 contacts aged ≥5 with known HIV infection. Only one visit was required to screen all eligible contacts for TB in 88 of 101 households (87%, 9 missing data).

### Step 4: TB evaluation of at-risk household contacts

Of the 131 at-risk contacts referred for medical evaluation, 26 contacts (20%, robust 95% confidence limits, 13%-29%) completed evaluation (range across health centers, 0%-32%). This included 17 of 59 (29%) symptomatic adults and older children, seven of 58 (12%) younger children, and two of 14 (14%) adults with known HIV infection.

### Contact investigation cascade

To summarize the four steps of the contact investigation cascade, (1) 61% of eligible index patients had a home visit scheduled; (2) 50% had a home visit initiated in which at least one possible contact was encountered; (3) 89% of eligible contacts completed screening for TB symptoms and risk factors; and (4) 20% of at-risk or symptomatic contacts completed TB evaluation. Therefore, the cumulative probability that a household contact with TB symptoms or risk factors would be screened and complete evaluation was 5%, the product of the conditional probability of completing each step (*i*.*e*., 61% x 50% x 89% x 20%).

The highest proportions of individuals lost were in Step 2 (completion of home visit, 50% drop-out) and Step 4 (completion of evaluation among contacts found to be at risk, 80% drop-out). The most common reasons for drop-out at Step 2 were that the index patient had left the household (10, 24%) or that the contacts canceled the home visit, saying they were too busy (10, 24%) (Table 3).

**Table 3:** Reasons for incomplete home visits.

Reason for incomplete home visit	Households
*n (%)*	*n = 42*^a^
Index patient left household	10 (24%)
Contacts reported being too busy	10 (24%)
Contacts refused	5 (12%)
Other^b^	5 (12%)
Index patient provided a wrong phone number	4 (10%)
Index patient reported being too busy	4 (10%)
Index patient changed mind	2 (5%)
Index patient died	2 (5%)

Legend:

^a^ This table describes a sequential sample of 42 index patients who completed the index patient interview and planned to have a home visit, but whose households were never visited.

^b^ Two were not followed up by lay health workers following an error with their tablets. One went to prison. One turned off his phone each time he saw the lay health worker calling. One decided not to tell the other members of the household.

### TB diagnoses and treatments

Five of the 26 (19%) patients completing evaluation were diagnosed with TB, including one adult by sputum smear examination, one adult by Xpert, one adult who was clinically diagnosed, and two younger children who were clinically diagnosed; all five were started on treatment. Four completed treatment and one died during the intensive phase. The yield of active TB cases as a result of contact investigation among all 287 eligible contacts was 1.7% (95% confidence interval, 0.6% to 4.0%), for a number of contacts needed to screen (NNS) of 58.

## Discussion

In recent years, active case-finding has received increased attention among TB control and elimination strategies because of its potential to expand detection of prevalent cases in high TB-burden settings and reduce incident cases by interrupting transmission.[12–17] To improve delivery of contact investigation and other active case-finding strategies, there is a need for attention to each step in the process to avoid excessive cumulative losses. We applied a cascade analysis to identify the greatest gaps in delivery of household contact investigation and follow-up evaluation in a programmatic setting. To our knowledge, no recent studies describe the contact investigation cascade in detail from identification of eligible index patients to evaluation of at-risk contacts.[18] We found that only about one-third of index patients are eligible for household contact investigation, and that because of drop-out from each of the key steps of the contact investigation cascade, in total only 5% of household contacts potentially at-risk for active TB were ever fully evaluated. This finding highlights the critical need for interventions to improve the delivery of contact investigation services in order to reach, identify, and treat undiagnosed TB patients in the community.

Of the four key steps in the contact investigation cascade, the greatest opportunities for improvement were at Step 1, Step 2, and Step 4. In Step 1 (scheduling of the home visit), consent to collect household information and carry out contact investigation was a major barrier. A significant proportion of index TB patients who were eligible for household contact investigation, 17% in total, did not consent. Another 22% of eligible index patients gave consent but left the clinic without providing sufficient information for scheduling the home visit. Given the variation between health centers in consent, we hypothesize that the rapport between lay health workers and index patients may contribute to index patients’ willingness to participate in household contact investigation. Further, the capacity of health centers to carry out routine contact investigation may vary by availability of personnel and transportation. Future research should investigate associations between index patient participation and health center and health worker characteristics in order to clarify the range of interventions that may be appropriate at this step.

At Step 2 (completion of home visit), reasons recorded for drop-out show that some index patients leave their households shortly after being diagnosed with TB, either to return to a home village to receive care from family, or because the diagnosis of TB precipitated conflict within the household. Lay health workers rely on index patients for help in physically locating households in dense urban communities without street names or addresses, and to facilitate the visit by introducing the health worker to their household contacts. Therefore, the departure of the index patient can be a major barrier to successful completion of the home visit. In an equal number of cases, the index patient facilitated introduction to household contacts, but the visit was canceled or repeatedly rescheduled because the household contacts said they were too busy. This suggests that some household contacts of TB patients are unmotivated to pursue screening for TB themselves, even when screening is offered at home. Future research should evaluate whether rapid-response home visits and/or providing small incentives for household participation in screening can reduce attrition at this step.

For Step 4 (completion of evaluation among contacts found to be at risk or symptomatic during the home visit), we hypothesize that the major barriers to completion are the cost and inconvenience of transport to the clinic, negative perceptions of clinics or clinic staff, and a lack of motivation. An intervention that reduces the need to visit the clinic itself could bypass all of these barriers. Moving forward, research in this area could test the effectiveness of interventions that extend evaluation services for TB into the household visit. Therefore, we are currently carrying out a household-randomized, controlled trial of an intervention offering home-initiated sputum collection during household contact investigation and short-messaging service (SMS) delivery of results (Pan-African Clinical Trials #201509000877140).

Our findings are consistent with a recent meta-analysis of TB-REACH-funded contact investigation activities in 11 high-burden countries, including Uganda, in which the proportion of index cases successfully screened varied from 3–91%, and the proportion of contacts successfully screened varied from 43–100%.[19] Our findings are also consistent with previous work demonstrating that even members of vulnerable populations in Uganda have access to mobile phones.[20–22] Only 5% of index patients and 1% of contacts reported that they did not have access to a mobile phone, a functional precondition of household contact investigation in low-income, urban settings where street addresses are uncommon.

In our study, only 1.7% of screened household contacts were diagnosed with TB, which is at the lower end for the yield of contact investigation in published epidemiological studies.[3,5] However, this yield is actually surprisingly high considering the high rates of drop-out among contacts referred for further evaluation: among contacts found to be symptomatic or at risk for TB and referred for further evaluation, 80% failed to complete that evaluation. Contacts who did not complete evaluation never had the opportunity to receive a TB diagnosis. This suggests that the yield of household contact investigation in this setting could be substantial if drop-out from the cascade were reduced.

Our study had several strengths. First, our cascade analysis complements a growing literature on barriers to uptake of contact investigation in programmatic settings[17,23,24], adding the most broad and detailed description to date of the TB contact investigation delivery cascade in a high-burden, low-income setting. Second, the prospective study design accounted for all index patients and household contacts as they actually moved through the evaluation cascade. Finally, all activities were carried out in collaboration with the National TB Program and its implementing partners in routine clinics by lay health workers already stationed at these health centers, following local policy for contact investigation. Our findings are therefore likely to be generalizable to programmatic conditions in the low-income settings where WHO currently recommends household contact investigation.

Our study also had certain limitations. First, while we sought to determine the effectiveness of routine contact investigation, our evaluation did not reflect fully programmatic conditions in a few, mostly minor, ways. In order to capture detailed data for each step in the cascade that is not available in TB registers, we required consent from index patients and their household contacts, and used tablet computers and fingerprint recognition. It is possible that consent procedures or the use of tablet computers and fingerprinting contributed to higher rates of refusal to participate. This could bias the estimate of the yield of household contact investigation compared to normal programmatic conditions. However, consent rates among contacts were very high, suggesting that index patients’ reticence may arise from concerns about contact investigation, not concerns about participating in research. Moreover, index patients who gave a reason for declining to participate most commonly cited time constraints, not a preference not to be involved in research.

Second, we note that household contacts of index cases who complete evaluation and initiate treatment for TB are likely to differ from household contacts of index cases who do not initiate treatment. Moreover, index patients who initiate treatment but drop out from the contact investigation cascade—and their contacts—may differ from those who persist. For example, having a coughing family member at home might motivate an index patient to facilitate household contact investigation. Future studies are needed to show that decreasing drop-out can increase TB case-finding, treatment initiation, and treatment completion.

### Conclusions and implications

These results emphasize the challenges of implementing comprehensive household contact investigation for TB in a routine setting, and identify the major sources of loss along the delivery cascade. Our findings suggest the greatest opportunities for improving the reach of TB contact investigation are in obtaining index patient permission for the home visit, detailed scheduling of the visit, and improving linkage to TB evaluation for symptomatic and at-risk contacts. Interventions designed to address key bottlenecks in this cascade are needed in order to realize the full potential of household contact investigation in these settings.

## Supporting information

S1 File Supporting information. This file contains supplemental description of methods and supporting tables.
(DOCX)

S2 File De-identified minimal data. This file contains the minimal data for recreating Figs 1 and 2 and Tables 1 and 2.
(XLSX)
