# Supplementary material for: Drop-out from the tuberculosis contact investigation cascade in a routine public health setting in urban Uganda: A prospective, multi-center study
Source: PLoS One. 2017 Nov 6;12(11):e0187145. doi: 10.1371/journal.pone.0187145 (PMC5673209; doi:10.1371/journal.pone.0187145)
Supplement: S1 File — This file contains supplemental description of methods and supporting tables. (DOCX) [file pone.0187145.s001.docx]

**SUPPORTING INFORMATION**

*Supplemental Methods*

A total of fourteen lay health workers screened all index patients for eligibility. Later, they screened and enrolled household contacts of participating index TB patients during home visits for treatment support. We provided additional training and support to these lay health workers for them to collect detailed data on each step in the contact investigation process. Four lay health worker supervisors oversaw these activities. In addition, four members of the study team visited lay health workers weekly to observe enrollment of index patients in the TB units and accompanied lay health workers on a selection of home visits to observe contact enrollment.

We employed fingerprint verification to prevent duplicate enrollment and verify follow-up visits across sites. By design, lay health workers verified children under age five by name because fingerprinting performs less reliably in this group.

*Register audit*

After completing data collection, we audited clinic registers at all sites, including the Possible TB Patient Register, the TB Lab Register, and the TB Treatment Register, to identify visits not captured by electronic data collection procedures. The audit identified only one patient visit not captured in study databases before data reconciliation.

**Table A. Characteristics of index TB patients whose households were reached and not reached for a home visit.**

| Characteristic ^a^ | Reached | Not Reached | p-Value |
| --- | --- | --- | --- |
| *n (%)* | *n=104^a^* | *n=103* |  |
| Men | 53 (56%) | 64 (62%) | 0.20 |
| HIV-seropositive | 29 (31%) | 41(40%) | 0.19 |
| Age groups |  |  |  |
| Adults (≥15 years) | 92 (97%) | 100 (97%) | 0.99 |
| Older children (5-14 years) | 2 (2%) | 1 (1%) | 0.56 |
| Young children (0-4 years) | 1 (1%) | 2 (2%) | 0.57 |
| >1 household | 6 (6%) | 0 (0%) | 0.01 |
| Number of contacts (range) |  |  |  |
| Reported | 3 (1 – 25) | 3 (1 – 12) | 0.05^b^ |
| Cough duration, weeks (25^th^-75^th^ %ile) | 8 (4 – 12) | 4 (3-12) | 0.61^b^ |
| Microbiologically confirmed | 94 (99%) | 102 (99%) | 0.99 |

Legend:

^a^Demographic and clinical data missing for 9 index patients whose households were reached.

^b^ Tests of counts performed using the Wilcoxon rank sum test.

**Table B. Cascade step proportion ranges by health center**

| Contact investigation step | Proportion range |
| --- | --- |
| (1) Home visit scheduled | 18% - 71% |
| (2) Home visit completed | 11% - 100% |
| (3) Contacts screened | 55% - 100% |
| (4) Contacts completing evaluation | 0% - 32% |

**Table C. Reasons provided by index patients for declining consent to participate.**

| Reason for declining | Index patients |
| --- | --- |
| *n (%)* | *n=28^a^* |
| Not enough time | 12 (42.9) |
| Other^b^ | 4 (14.3) |
| Don’t trust study | 3 (10.7) |
| Not benefiting | 2 (7.1) |
| Worried about risks | 2 (7.1) |
| Seek permission | 2 (7.1) |
| Feel too sick | 2 (7.1) |
| Worried about reputation | 1 (3.6) |

Legend:

^a^ This table provides data on 28 consecutive patients among those who declined consent; the other patients who declined consent were enrolled before this question was added to the protocol.

^b^ Two preferred “to do at home.” One wanted “to talk to her husband first.” The final declining respondent “needed some time.”
